# Late Diagnostic Clues in Rapidly Progressing Probable Heidenhain Variant of Creutzfeldt–Jakob Disease

**DOI:** 10.1155/crnm/4618310

**Published:** 2024-11-25

**Authors:** Rahul Gaini, Julia Denniss, Elijah Lackey

**Affiliations:** ^1^Department of Neurology, Duke University Hospital, Durham, North Carolina, USA; ^2^Department of Neurology, The Johns Hopkins Hospital, Baltimore, Maryland, USA

## Abstract

Presenting symptoms of sporadic Creutzfeldt–Jakob disease (sCJD) are variable, and as imaging and EEG may be normal in the early to middle stages of the disease process, serial testing is vital when there is clinical suspicion for sCJD. We present a case of probable Heidenhain variant of sCJD (HvCJD) with notable rapid progression. A 72-year-old woman presented with neurological decline following new–onset visual changes. Over the course of 3 weeks, she developed ataxia followed by paranoia, memory impairment, and visual hallucinations. An extensive workup from 1 week prior at an outside hospital was unrevealing and included two magnetic resonance imaging (MRI) studies read as normal and an EEG without periodic sharp wave complexes. Repeat of imaging at our hospital showed cortical restricted diffusion in the right occipital lobe. In combination with new periodic sharp wave complexes visualized on prolonged EEG, concern was raised for sCJD. Palliative care was consulted early in the hospitalization, and the patient was transitioned to comfort care and discharged 3 days after admission. She declined quickly and passed away at home within a week, one day before her send out CSF sample resulted with a positive real-time quaking-induced conversion (RT-QuiC) and markedly elevated T-tau protein and 14-3-3 gamma. As there is no treatment for this fatal disease, palliative engagement and discussion of goals of care in cases of CJD is critical in providing compassionate care for the patient and their family. High clinical suspicion warrants discussion of comfort care measures even prior to confirmation with RT-QuiC.

## 1. Introduction

**Figure 1:**
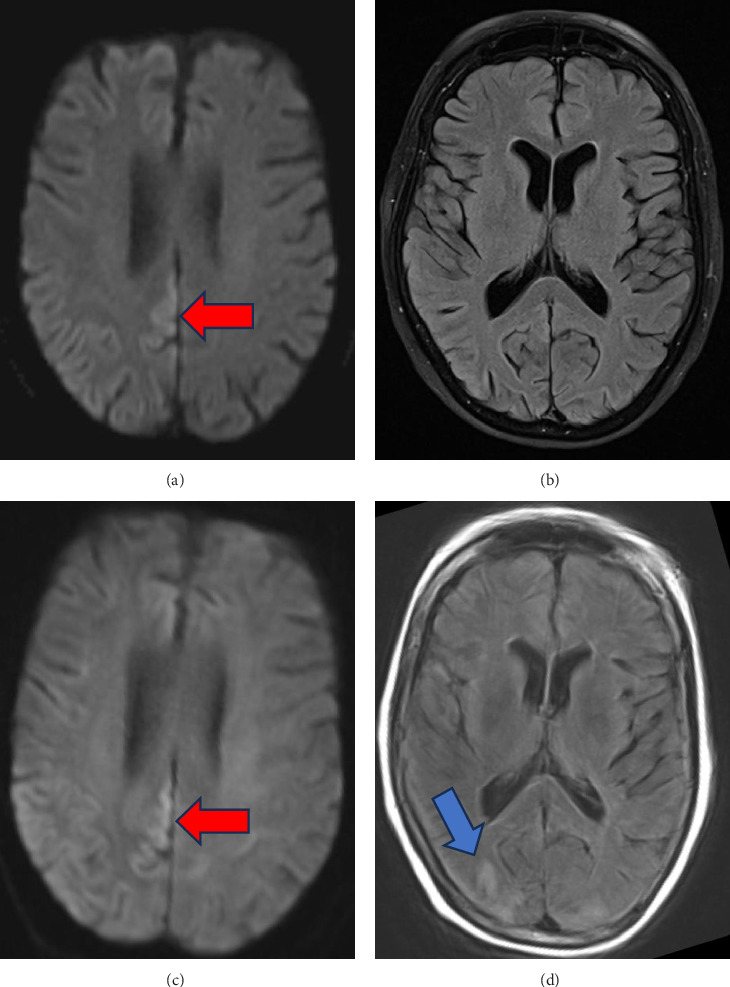
Magnetic resonance imaging of the patient's brain with and without contrast. (a) and (b) were obtained on the second week of symptoms at an outside hospital. (a) Diffusion weighted imaging (DWI) showing restricted diffusion most prominent in the right occipital lobe (red arrow). (b) T2/FLAIR sequence showing minimal FLAIR changes in that region. (c) and (d) were obtained on the third week of symptoms. (c) the DWI scan again showing diffusion restriction in the occipital lobe. In (d), progression of FLAIR signal in the right occipital lobe (blue arrow) can be observed on the T2/FLAIR sequence compared with (b).

Creutzfeldt–Jakob disease (CJD) is a prion disease whose pathogenesis involves invasion of protease-resistant abnormally shaped proteins, known as prions, through sporadic, familial, or infectious means. These prions act as templates for the generation of additional endogenous prions through acquisition of high *β*-sheet content, leading to conversion of native protein (PrP^C^) to modified protein (PrP^Sc^) and causing rapid spread and clinical decompensation [1, 2]. The incidence of sporadic CJD (sCJD) falls between one and two cases per million population, though recent data suggest an increased incidence within some countries [3]. Unfortunately, a prognosis from onset of clinical symptoms is on the order of months [4], and CJD is uniformly fatal as there is no effective treatment [5]. In the Heidenhain variant of sCJD (HvCJD), visual disturbances can be the presenting symptom due to propensity of prions towards the occipital cortex, reflected by preferential diffusion restriction and T2-weighted FLAIR changes in this part of the cortex on magnetic resonance imaging (MRI) sequences (Figure 1) [6].

## 2. Case Presentation

A 72-year-old female with medical history of hypertension, hypothyroidism, type 2 diabetes mellitus, and melanoma presented as an outside hospital transfer for further management of neurologic decline over three weeks. The patient initially presented with visual disturbances. Her husband first had concerns when she had trouble positioning her signature on the correct line at an outpatient appointment. Within days, she developed trouble seeing objects and began skipping lines when reading. One week thereafter, she developed a wobbly, wide-based gait with left hemibody weakness and visual hallucinations, paranoia, confusion, and trouble with short-term memory. Symptoms gradually worsened over two more weeks to the point that the patient was completely dependent on her husband for activities of daily living. She was previously fully independent, even gardening daily and helping her husband with his medications. She had no recent travel outside of the country, drug use, known toxic exposures, changes in diet, or anything else out of the ordinary. There were no recent vaccinations, medication changes, or other identifiable iatrogenic causes of symptoms. There was no known history of similar symptoms in family members. She was seen at two outside hospitals with extensive neurologic testing including two MRI studies of the brain, one with contrast, lumbar puncture with protein, cell count, glucose, meningitis polymerase chain reaction (PCR), cultures, Lyme disease, Rocky Mountain spotted fever, and electroencephalogram (EEG). Workup was determined to be unremarkable aside from concern for previous epileptic activity on EEG, and the patient was subsequently started on lacosamide followed by levetiracetam later in her course. On discharge, she was still providing her own history and able to hold a conversation, though her family had noticed irritability and mood changes. Her husband then brought her to our hospital 4 days later for another opinion given ongoing decline even with anti-seizure medications.

On arrival at our emergency department (Day 1), the patient was vitally stable. Exam revealed a woman who was not alert but easily arousable to voice and minor stimulation. Attention and concentration were poor, but she was able to follow simple commands. Speech was dysarthric and limited to single-word responses. Cranial nerve exam revealed a left lower facial droop with flattening of the nasolabial fold. Muscle tone was increased with marked rigidity throughout, notably more on the left hemibody compared to the right. Hyperreflexia (3+) was observed diffusely and symmetrically. Exaggerated startle response was absent. The patient was unable to reliably comply with strength, sensory, and coordination testing, but no obvious deficits were appreciated. Gait testing was deferred due to fall risk.

**Figure 2:**
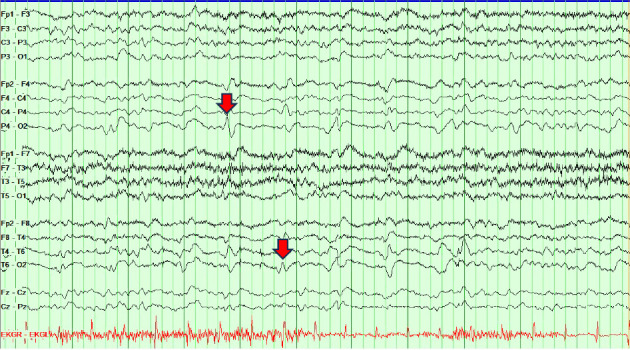
Electroencephalogram (EEG) completed at approximately Week 4 of symptoms. The background was diffusely slow in the theta range with focal slowing over the right hemisphere. Also present over the right hemisphere were waxing and waning lateralized periodic sharp wave discharges at about 1 Hz consistent with CJD.

Given the time course and symptoms, the differential included a vascular insult (vasculitis, multiple ischemic infarcts, and posterior reversible encephalopathy syndrome), rapidly progressive degenerative process (CJD, normal pressure hydrocephalus, and dementia variant), toxic/metabolic insult (Wernicke–Korsakoff syndrome and neuroleptic malignant syndrome), central nervous system infection, and inflammatory processes. On Day 1, initial workup of complete metabolic panel, complete blood counts, urinalysis, inflammatory markers, and thiamine level was not clinically significant. Further serum and cerebrospinal fluid (CSF) studies were obtained next. Serum autoimmune encephalopathy panel was negative. CSF studies showed mildly elevated protein at 54, glucose of 127, zero nucleated cells, and negative autoimmune encephalopathy panel, fungal and bacterial cultures, meningitis PCR, and VDRL studies. A tube of CSF was sent on ice to the National Prion Disease Center to test for CJD. Repeated MRI of the brain with and without contrast showed gyriform cortical–restricted diffusion along the right occipital lobe with areas of T2/FLAIR signal in the bilateral occipital lobes (Figure 1(b)). Prolonged EEG was notable for intermittent periodic sharp wave complexes at 1 Hz in the right-sided posterior leads (Figure 2). Based on these findings, our clinical suspicion for HvCJD was very high. On Day 2, our palliative care colleagues were consulted, and our patient was transitioned to comfort care as per the family's wishes. The patient was discharged on Day 3. On Day 9, she passed away peacefully at home. On Day 10, the patient's send out CSF sample resulted with a positive RT-QuiC along with markedly elevated T-tau protein and 14-3-3 gamma, confirming the diagnosis of CJD.

## 3. Discussion

Initial clinical symptoms of sCJD are often nonspecific and variable. Presenting symptoms include cognitive decline (70%), ataxia (39%), visual signs (27%), psychiatric manifestations (23%), and aphasia (23%) [1]. In our case, the patient's preliminary symptom was changes in her vision, suspected to be visual field cut or decline in visual acuity based on the husband's description. Cognitive decline and psychiatric symptoms did not manifest until the following week. She also had two normal MRI's of the brain, one with contrast, along with lack of reported periodic sharp wave complexes on previous EEGs before transfer to our hospital (Figure 1(c)). Nonetheless, there were a few distinguishing features throughout her course that triggered suspicion for CJD. For one, our patient had a rapid, severe, subacute decline without evidence of other etiologies on lumbar puncture and MRI. In addition, imaging and EEG findings in the early stages of the disease can also be normal, and serial testing is necessary, given that many characteristic elements may only arise in the middle and late stages of the disease [1].

The third MRI of the patient's brain ultimately revealed cortical diffusion restriction and T2 FLAIR changes in the right occipital cortex (Figure 1), consistent with cortical hyperintensity which can be seen in CJD [7]. In reviewing outside hospital imaging, cortical diffusion restriction in the right occipital lobe was present, though the FLAIR signal was less apparent (Figures 1(a) and 1(b)). We speculate that such changes may have been missed or attributed to seizures or bone artifact. A repeat EEG at our hospital also presented positive diagnostic evidence for CJD with periodic 1 Hz sharp wave complexes in the posterior right-sided leads (Figure 2). When evaluating an EEG for possible CJD, the Steinhoff criteria can be applied to improve specificity by assessing for sharp wave complexes with duration between 100 and 600 ms, intercomplex intervals between 500 and 2000 ms, and at least five repetitive intervals with a duration difference of 500 ms [8]. Though not pursued in this case, genetic testing can be sent to assess for genotypic polymorphisms at codon 129 and/or presence of point or insert mutations of the prion protein gene [9]. To confirm diagnosis, CSF studies provide good sensitivity for the disease. While the CSF 14-3-3 test and tau test have poor specificity (28% and 67%, respectively) [10], the RT-QuiC has shown to have a specificity of 99%-100% [11]. However, these studies require send out to the National Prion Center for processing, which takes time. Our patient passed away prior to confirming the diagnosis by RT-QuiC, within just four to five weeks of symptom onset, which is strikingly more rapid than the mean illness duration of 4 months in reported cases of sCJD with isolated visual symptoms at onset [12]. Therefore, early recognition of the disease via clinical symptoms and other diagnostics is key to allow for timely palliative engagement and pursuance of comfort care measures, thereby providing humanistic care for the patient and their family.

## Data Availability

The data that support the findings of this study are available from the corresponding author upon reasonable request.
